# Fast and precise targeting of single tumor cells *in vivo* by multimodal correlative microscopy

**DOI:** 10.1242/jcs.181842

**Published:** 2016-01-15

**Authors:** Matthia A. Karreman, Luc Mercier, Nicole L. Schieber, Gergely Solecki, Guillaume Allio, Frank Winkler, Bernhard Ruthensteiner, Jacky G. Goetz, Yannick Schwab

**Affiliations:** 1 Cell Biology and Biophysics Unit, European Molecular Biology Laboratory, Heidelberg 69117, Germany; 2 MN3T, Inserm U1109, Strasbourg 67200, France; 3 Université de Strasbourg, Strasbourg 67000, France; 4 LabEx Medalis, Université de Strasbourg, Strasbourg 67000, France; 5 Fédération de Médecine Translationnelle de Strasbourg (FMTS), Université de Strasbourg, Strasbourg 67000, France; 6 Department of Neurooncology, University Hospital Heidelberg, Heidelberg 69120, Germany; 7 Clinical Cooperation Unit Neurooncology, German Cancer Research Center (DKFZ), Heidelberg 69120, Germany; 8 Evertebrata Varia, Zoologische Staatssammlung München, Munich 81247, Germany

**Keywords:** Electron microscopy, Correlative microscopy, Extravasation, Intravital imaging, Invasion, Metastasis

## Abstract

Intravital microscopy provides dynamic understanding of multiple cell biological processes, but its limited resolution has so far precluded structural analysis. Because it is difficult to capture rare and transient events, only a few attempts have been made to observe specific developmental and pathological processes in animal models using electron microscopy. The multimodal correlative approach that we propose here combines intravital microscopy, microscopic X-ray computed tomography and three-dimensional electron microscopy. It enables a rapid (c.a. 2 weeks) and accurate (<5 µm) correlation of functional imaging to ultrastructural analysis of single cells in a relevant context. We demonstrate the power of our approach by capturing single tumor cells in the vasculature of the cerebral cortex and in subcutaneous tumors, providing unique insights into metastatic events. Providing a significantly improved throughput, our workflow enables multiple sampling, a prerequisite for making correlative imaging a relevant tool to study cell biology *in vivo*. Owing to the versatility of this workflow, we envision broad applications in various fields of biological research, such as cancer or developmental biology.

## Abstract (precis)

**Highlighted Article:** We provide here a novel correlative workflow combining intravital microscopy, microCT and 3D electron microscopy to reveal metastatic events in mouse brain and skin tissue at high resolution.

## INTRODUCTION

Metastasis relies on a series of steps, including invasion of the tissue and circulation through the vasculature to reach a secondary distant site (Valastyan and Weinberg, 2011). Although relevant *in vitro* models have been created to study these processes (Gligorijevic et al., 2014), they have failed so far to recapitulate the complexity of living tissues. Intravital microscopy (IVM) of invasive tumor cells has enabled *in vivo* studies of the metastatic cascade (Gligorijevic et al., 2014; Kienast et al., 2010). Here, tumor progression can be imaged in various animal models upon, for example, orthotopic, subcutaneous or intra-circulation injection of tumor cells (Karreman et al., 2014; Leong et al., 2014; Sahai, 2007; Stoletov et al., 2010). For that purpose, implementation of an imaging window allows for long-term deep-tissue monitoring of invasive behavior of tumor cells in living animals (Alexander et al., 2008; Beerling et al., 2011; Gligorijevic et al., 2014; Ritsma et al., 2013). We, and others, have successfully studied key steps of extravasation by performing IVM through a cranial window (Kienast et al., 2010). Extravasation is a crucial, yet rare and inefficient step in metastasis, which makes it difficult to study *in vivo*. There is, for example, no direct evidence as to how tumor cells arrest, adhere to the endothelium wall and cross the blood–brain barrier *in vivo* (Reymond et al., 2013). In addition, tumor cells use distinct mechanisms for invading the neighboring tissue (Friedl and Alexander, 2011). Understanding how cytoskeletal behavior, cell adhesion and proteolytic activity are integrated *in vivo* requires studying these events at the scale of a single cell, within its pathological context. IVM can capture dynamic metastatic events, but its resolution is insufficient to reveal subcellular events or the interactions of tumor cells with the surrounding tissue.

Correlating functional IVM to three-dimensional electron microscopy (3DEM) carries great potential in revealing the *in vivo* features of patho-physiological processes at nanometer resolution. The power of combining these imaging techniques is well established (Bishop et al., 2011; Briggman and Bock, 2012; Durdu et al., 2014; Goetz et al., 2014; Kolotuev et al., 2010; Maco et al., 2013). Because of a low throughput however (Karreman et al., 2014), intravital correlative microscopy has failed to provide the quantitative sampling needed for translational research.

**Fig. 1.**
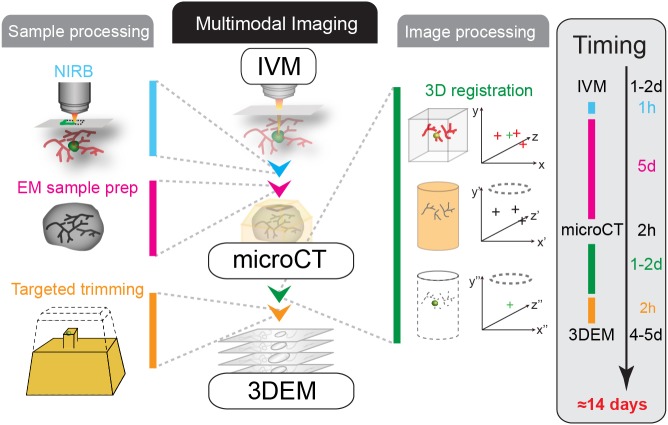
**Workflow for multimodal correlative microscopy.** Multimodal imaging of metastatic events observed *in vivo* requires specific sample and image processing methods. First, the event of interest is captured by using IVM (time, ∼1–2 days). The position of the ROI is marked at the tissue surface with NIRB (1 h). Based on this macroscopic mark, a biopsy containing the ROI is dissected and processed for electron microscopy analysis (1 day+4 days). The resin-embedded sample is then imaged with microCT (2 h). The imaged volume obtained from the IVM is registered to the microCT volume by matching correlated pairs of landmarks in Amira software (1–2 days). 3D registration allows determination of the position of the resin-embedded ROI relative to the surface of the block. The resin block is accurately trimmed to expose the tumor cell for electron microscopy imaging (2 h). Finally, 3DEM of the ROI is performed (4–5 days). If all the steps are performed without interruption, the average duration of this workflow is thus roughly 2 weeks.

The main bottleneck for intravital correlative microscopy is retrieving single objects in the electron-microscopy-processed sample. Unfortunately, processing tissue for 3DEM generally results in a loss of fluorescent signal, prohibiting the use of fluorescence microscopy to determine the position of the region of interest (ROI) in the volume of the electron microscopy sample. Moreover, the major sample distortions that result from fixation and resin embedding complicate the registration of the IVM into the electron microscopy datasets (Karreman et al., 2014). As a result, the targeted volume needs to be retrieved by correlating native or artificial landmarks that are encountered when serial-sectioning the sample, which in our experience (Karreman et al., 2014), can easily take more than 3 months. Moreover, such an approach is limited to relatively thin tissue samples, such as brain slices (Bishop et al., 2011; Maco et al., 2013) or skin (Karreman et al., 2014). Collecting quantitative electron microscopy data on multiple metastatic events *in vivo* therefore requires new strategies, endowed with an enhanced throughput. Here, we describe a novel method that exploits microscopic X-ray computed tomography (microCT) to precisely correlate the IVM volume with the electron-microscopy-processed resin-embedded sample, enabling the move from *in vivo* imaging to 3DEM within two weeks (Fig. 1). We developed and applied this approach to study single tumor cells that had been xenografted into a living mouse, showing the potential of this method to reveal key aspects of the plasticity and complexity of tumor cell invasion and metastasis. The versatility of this workflow is expected to enable a large range of applications in biology.

## RESULTS

### Intravital microscopy of metastasizing cells in the mouse brain cortex vasculature

**Fig. 2.**
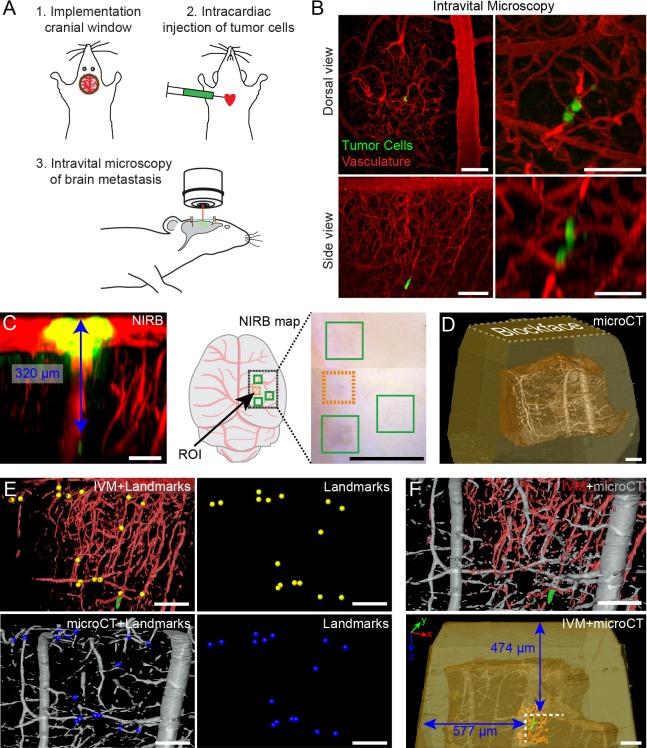
**IVM and microCT imaging of tumor cells in the mouse brain cortex, followed by 3D registration of both datasets.** (A) A chronic cranial window is implanted 3 weeks before intravital imaging is performed. JIMT1 GFP-expressing cells are injected into the left ventricle of the mouse heart. For IVM analysis, the cranial window is mounted onto a customized stage that allows reproducible orientation, and thus imaging, of the same ROI over multiple days. (B) IVM analysis of an arrested tumor cell in the brain cortex vasculature. Dorsal views (top panels), *z*-projection of a large field of view around the ROI at 2 days post injection (top left panel) and of a smaller field of view, imaged 3 days post injection (top right panel). Side views (bottom panels), *x*,*z*-projection of the ROI, 2 days (left bottom panel) and 3 days (right bottom panel) post injection. Scale bars: 100 μm in left panels, 50 μm in right panels. (C) After IVM acquisition and perfusion fixation, the position of the ROI is marked by using NIRB at the surface of the brain, producing autofluorescence in both the green and red channel (yellow). The *x*,*z* projection shows the vasculature (red) and how the NIRB landmark is confined to the surface of the brain, distant (blue arrow) to the tumor cell (green). A cartoon and an image of the mouse brain show the relative positions of several NIRB landmarks. Aside from the landmark positioned above the ROI (orange box), three additional marks were created as references (green boxes) to facilitate targeting of the selected ROIs when dissecting the biopsies. Scale bars: 100 μm (left panel), 1 mm (right panel). (D) The microCT dataset shows the tissue biopsy (brown) within the resin block (yellow) and the blood vessels (gray). Scale bar: 100 μm. (E) 3D registration of the vasculature as segmented from the two imaging modalities – IVM (red) and microCT (gray). Corresponding points in both datasets are located and marked (yellow spheres for IVM and blue spheres for microCT). Scale bars: 100 μm. (F) Based on the landmarks shown in E, the IVM volume is registered into the microCT dataset with Amira software, which enables precise determination of the position of the tumor cell (green) within the resin block and relative to its surface (bottom panel in F). Scale bars: 100 μm.

Correlative imaging of the initial steps of tumor cell extravasation was performed on our established mouse model, in which we could reliably identify fluorescently labeled metastasizing tumor cells and track them over time. Upon intra-cardiac injection of GFP-labeled HER2-positive breast cancer cells (JIMT1), IVM analysis of the brain cortex was performed through a cranial window (Fig. 1, Fig. 2A). We labeled the blood vessel network with fluorescent dextran to create a stable 3D reference map of the area around the tumor cell. Tumor cells were arrested through physical occlusion in vessel bifurcations before extravasation (Kienast et al., 2010) and could be tracked over days by using IVM (Fig. 2B; Movie 1). IVM imaging of the volume allows recording of the position of the tumor cell relative to the local vasculature at the ROI. At a later stage in the workflow, the complex vessel network is exploited as a 3D roadmap to retrieve the tumor cell with 3DEM.

After fixation using intra-cardiac perfusion, the position of the ROI was immediately marked at the cortex surface using near-infrared branding (NIRB) (Bishop et al., 2011; Karreman et al., 2014; Maco et al., 2013) (Fig. 2C; Movie 1), enabling precise dissection of a small biopsy (<1 mm^3^) containing the ROI. The sample was then processed for electron microscopy analysis and embedded in resin. This shows that we can reliably track metastatic events in the brain vasculature and, based on NIRB marking, biopsy the imaged region after fixation.

### Retrieval of the tumor cell following electron microscopy processing

The high X-ray attenuation of the heavy metals used during electron microscopy processing (i.e. osmium tetroxide and uranyl acetate) enables the imaging of the resin-embedded sample with microCT (Bushong et al., 2015; Handschuh et al., 2013; Keene et al., 2014; Metscher, 2009; Sengle et al., 2013). MicroCT volumes of the samples were rendered in 3D to display the resin block surface, the embedded tissue and its vascular network (Fig. 2D; Fig. S1). The models of the vascular network obtained from the IVM and the microCT datasets were then processed for non-linear 3D registration (Fig. 2E; Movie 2). Corresponding structural features, visible in both datasets, were used to seed pairs of landmarks (Fig. 2E, yellow and blue spheres) and were overlaid in 3D (see Materials and Methods for further details). Coarse 3D registration between the IVM (red) and the microCT (gray) volumes (Fig. 2E) was followed by fine registration using higher magnification IVM acquisitions (Fig. S2). Docking of the IVM model into the microCT dataset then enabled us to predict the position of the tumor cell, visible in the green IVM channel, within the resin block (Fig. 2F; Movie 2).

Knowing the distance between the block surface and the predicted position of the tumor cell allowed accurate block trimming to rapidly approach the ROI (Fig. 2F, bottom panel). This efficiently enabled us to expose the targeted cell to focused ion beam scanning electron microscopy (FIB-SEM). Because FIB-SEM can only effectively mill a depth of up to 60–90 µm into the resin sample, we trimmed the flank of the block (perpendicular to the FIB beam) to ∼20 µm from the targeted tumor cell. Similarly, the distance between the block face and the ROI (Fig. 2D,F) should be trimmed to less than 5 µm (Bushong et al., 2015) to prevent unnecessary long milling times before reaching the cell of interest (Movie 3). To target the ROI with such precision, we collected a few thick sections as intermediate ‘check points’ when manually trimming to the targeted depth (Fig. S3). These sections were inspected by using light microscopy and compared to virtual sections extracted from the microCT dataset, thereby assessing the exact progression of the trimming. For trimming depths of around 500 µm, 3–5 check points were sufficient to correct for potential trimming inaccuracy (estimated to be between 5 and 10%, compared to the nominal settings of the ultramicrotome) and allowed a targeting precision of <5 µm. Thus, using microCT-based registration, we could accurately target a cell of interest within a millimeter-thick resin-embedded tissue.

### 3DEM of metastasizing cells arrested in the brain vasculature

**Fig. 3.**
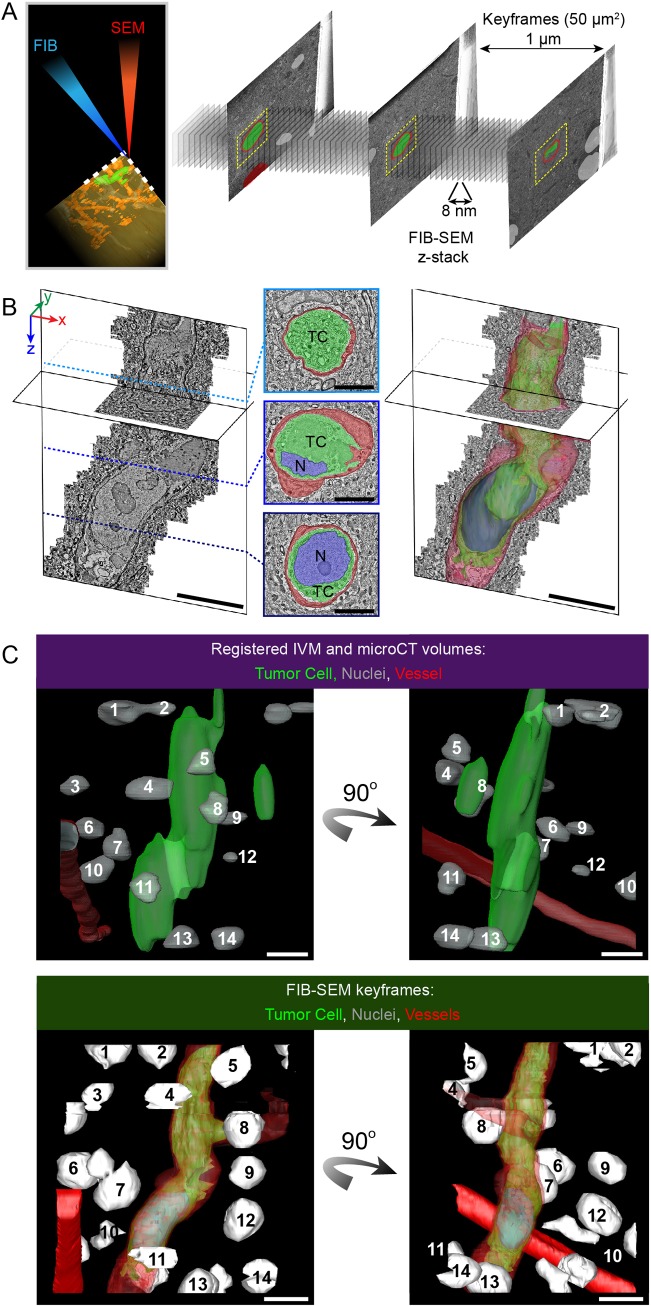
**FIB-SEM imaging of a tumor cell arrested in the vasculature of the brain.** (A) Automated 3DEM. The resin block was precisely trimmed to expose the tumor cell for acquisition of data using FIB-SEM. During the course of the FIB-SEM data acquisition, low-magnification ‘key frames’ were obtained (*z* spacing ∼1 μm, size 50×50 μm). (B) The FIB-SEM *z*-stack (6350 frames, 8 nm isotropic voxel size) was used to study the fine organization of the tumor cell within the blood capillary. Frames of the FIB-SEM acquisition were segmented to show the tumor cell (TC, green), its nucleus (N, blue) and the capillary's endothelial cells and basal lamina (red). Scale bars: 10 μm (3D models), 5 μm (segmented sections). (C) Multimodal correlation – combining IVM, microCT and electron microscopy imaging to retrieve the tumor cell. Top panel, docking the IVM model in the microCT dataset enabled us to predict the position of the tumor cell (green, from IVM), relative to the nuclei (light gray, from microCT) and to a blood vessel (red, microCT). The nuclei and the vessel could be detected in the microCT scan because of their lower density relative to the surrounding tissue. Bottom panel, segmentation of the FIB-SEM key frames results in a 3D model of the nuclei (white), the vessels (red) and the tumor cell (green). Two views of the models are shown, rotated 90° with respect to each other. Comparing both models enabled us to correlate the positions of the nuclei that were visible with microCT and electron microscopy imaging. Matching nuclei are numbered in the top and bottom panels. Scale bars: 10 μm.

We used FIB-SEM to collect 6350 serial images (8 nm pixel size in the *x*,*y* plane, and 8 nm milling depth in the *z* plane) of the tumor cell within the blood capillary (Fig. 3A,B; Movie 4). In addition, we acquired key frames (Fig. 3A) every 1 µm (50×50 µm^2^, pixel size 24.4 nm, acquired every 1 µm) to obtain low-magnification overviews of the ROI showing surrounding cell nuclei and blood vessels (Narayan et al., 2014). To illustrate the efficiency of the multimodal correlation, the predicted position of the tumor cell within the resin block (as determined by registering the IVM volume into the microCT volume; Fig. 3C, top panel) was compared to the manual segmentation of the key-frame-volume (Fig. 3C, bottom panel).

**Fig. 4.**
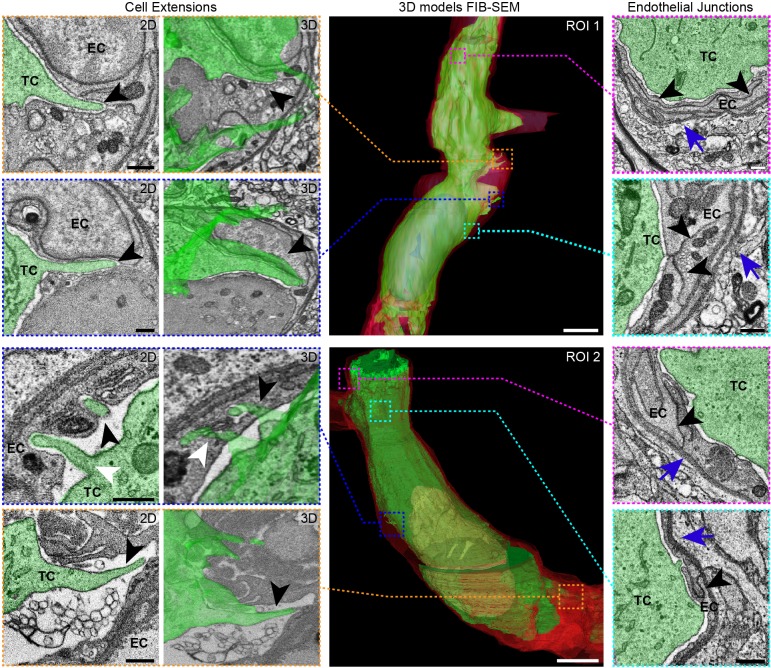
**3DEM imaging of an arrested tumor cell in brain capillaries.** 3DEM analysis reveals tumor cell (TC) extensions pointing towards and into the endothelial cell layer (EC) (first and second columns from the left, 2D and 3D views of the cell extensions, respectively; indicated with an arrowhead). Electron microscopy analyses revealed the junctions between endothelial cells (right-most column, arrowheads) and the basal lamina (right column, blue arrows). These ultrastructural features were observed in the FIB-SEM *z*-stacks obtained from two different tumor cells that were arrested in the vasculature of the brain (ROI1 and ROI2). The dotted lines and boxes in the 3D models of the FIB-SEM *z*-stack indicate from which regions of the tumor cells these images were obtained. Scale bars: 500 nm (electron microscopy images); 5 μm (3D models).

**Fig. 5.**
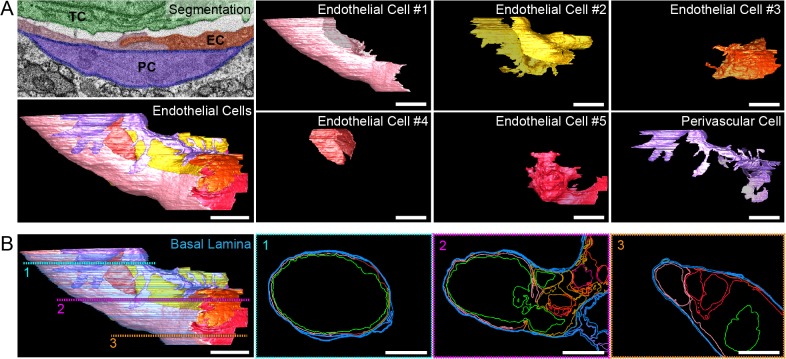
**3DEM imaging allows dissection of the complex structural organization of the vessel containing an arrested tumor cell.** (A) Each individual cell lining the tumor-cell-containing vessel, as well as the basal lamina and a perivascular cell we segmented. In the top left image (‘segmentation’), a detailed image of a FIB-SEM slice is shown where two neighboring endothelial cells (EC, pink and red), a perivascular cell (PC, purple), the basal lamina (blue) and the tumor cell (TC, green) are segmented. The bottom left panel shows a 3D representation of all the cells lining the vessel that contains the lower part of the ROI2 tumor cell (Fig. 4). In the columns and rows on the right side, each cell is shown individually. Scale bar: 5 μm. (B) A 3D representation of the basal lamina (blue) is shown (left panel), together with the plasma membranes of endothelial cells (various colors), of the perivascular cell (purple) and the lower part of the ROI2 tumor cell (green; Fig. 4). The three panels on the right depict different sections through the model that reveal the contours of the basal lamina, the endothelial cells and the tumor cell. At the level of the vessel bifurcation, the organization of the endothelial cells and of the basal lamina displays complexity, highlighting potential endothelial remodeling in this area. Scale bar: 5 μm.

A second tumor cell, observed in a different region of the same brain, was also targeted and imaged with FIB-SEM (Fig. S4, Movie 5), demonstrating the reproducibility of our approach. Cell extensions were found to either intercalate between or to invaginate into endothelial cells of the capillary (Fig. 4; Movies 4 and 5). In addition to elucidating the ultrastructure of the tumor cell, this technique also gives access to the fine organization of the capillary, the parenchyme and the surrounding tissue (Fig. 5A; Movie 6). It therefore enables the study of potential alterations to the microenvironment of an arrested tumor cell that is initiating extravasation (Fig. 5A,B; Movie 6). Being able to systematically and repeatedly collect such data from multiple experiments will lead to a better understanding of the key steps of the metastasis cascade.

### Intravital imaging of an invasive subcutaneous tumor xenograft

**Fig. 6.**
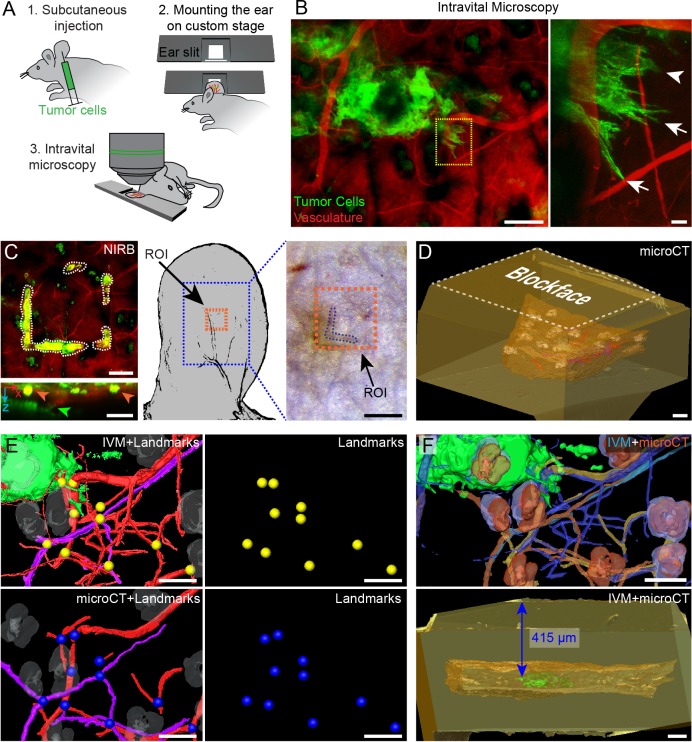
**IVM imaging of tumor cell protrusions of subcutaneous xenografts.** (A) D2A1 LifeAct-YPet-expressing cells were injected subcutaneously into the mouse ear. After 2 weeks of tumor growth, the mouse was anesthetized and positioned on a custom-built stage. The ear slit in the stage kept the ear flat and still during intravital imaging. (B) *z*-projection of the IVM *z*-stack showing the tumor mass (Ypet, green), its invasive front and the vasculature (Evans Blue, red). The right panel shows a magnified view of the area boxed in the left panel and reveals invasive cells with distinct morphologies. Two cells have extended protrusions (arrows), whereas another one has smoother contours (arrowhead). Scale bars: 100 μm (left panel); 10 μm (right panel). (C) After IVM analysis, the area of interest was marked by using NIRB (white dotted line) at the skin surface. Bottom panel, *x*,*z* projection of the *z*-stack. The green arrowhead points to a tumor cell protrusion. The NIRB markings (orange arrowheads) are confined to the surface of the skin and are distant from the invasive front of the tumor mass. Following perfusion fixation, the NIRB markings (orange box) remain visible on the skin biopsy. Scale bars: 100 μm (left panels); 50 μm (right panel). (D) The resin-embedded sample was scanned by using microCT. The microCT dataset shows the skin tissue biopsy (brown), with the hair follicles (gray), nerves (purple) and blood vessels (red), within the resin block (yellow). Scale bar: 100 μm. (E) 3D registration of the tissue features as segmented from the two imaging modalities – IVM (top left panel) and microCT (bottom left panel). Corresponding points in both datasets were located and marked (yellow spheres for IVM and blue spheres for microCT, left and middle panels). Scale bars: 100 μm. (F) Top panel, based on the reference points shown in E, the IVM model (shown in blue and green) is registered to the microCT dataset (yellow and orange), which enabled us to determine the position of the ROI inside the resin block (bottom panel) and relative to the block surface (415 µm). Scale bars: 100 μm.

Unraveling the mechanisms and plasticity of metastatic pathways demands the establishment of a technology that enables the study of each step – from primary tumor invasion to metastasis formation. To validate its versatility, we decided to apply our approach to another *in vivo* model designed to track primary tumor invasion – invasive tumor xenografts in the mouse ear (Karreman et al., 2014). In this model, highly invasive fluorescent tumor cells (Shibue et al., 2013) are injected subcutaneously (Karreman et al., 2014). IVM revealed actin-rich protrusions at the edge of the tumor mass (Fig. 6A,B; Movie 7), and invasive regions of the tumor were recorded with time-lapse imaging (data not shown). We detected invasive cells with visible morphological differences in the same invasive front (Fig. 6B), confirming that our model can recapitulate the established adaptability of tumor cell invasion (Friedl and Alexander, 2011). To analyze the ultrastructure of these invasive cells, we fixed the tissue and performed NIRB above the protrusions to mark the position of the ROI at the ear surface (Fig. 6C). The NIRB mark was then used to retrieve the ROI before further electron microscopy processing and resin embedding, which demonstrates that we could safely retrieve IVM-imaged ROIs from multiple tissues (brain and ear).

### Targeting protrusions of invading tumor cells in the electron microscopy processed sample

**Fig. 7.**
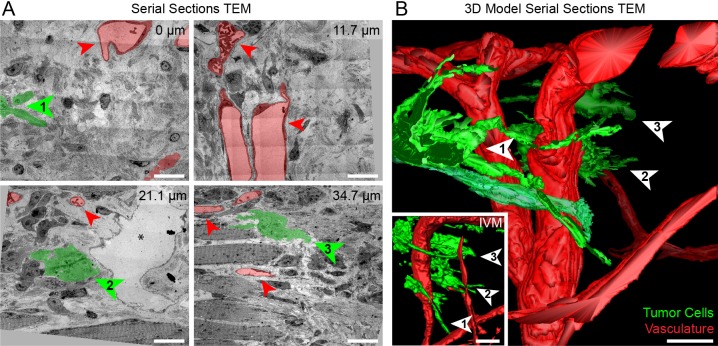
**Serial TEM imaging and correlation of tumor cell protrusions in skin tissue.** (A) Large fields of view comprising stitched TEM micrographs from 130-nm thick sections of the volume of interest shown in Fig. 6. The blood vessels (red) and tumor cells (green) are highlighted. Four different depths (marked in μm relative to the first selected section) are shown to illustrate specific ultrastructural hallmarks of the tumor cells and their microenvironment. Three cells from the invasive front imaged with IVM have been retrieved and highlighted (numbered arrowheads). The electron-lucent area in the lower left panel, marked with an asterisk, is a lymphatic vessel. Scale bars: 20 μm. (B) 3D model of the tumor cell protrusions (green) and vessels (red), segmented from 330 serial TEM sections. Corresponding tumor cell protrusions in A and B are indicated with numbered arrowheads. 3D models and TEM images of the cells indicated with arrowheads 1 and 3 are shown in Fig. 8. The inset shows a 3D model of the same region, imaged by using IVM. Scale bars: 20 μm.

**Fig. 8.**
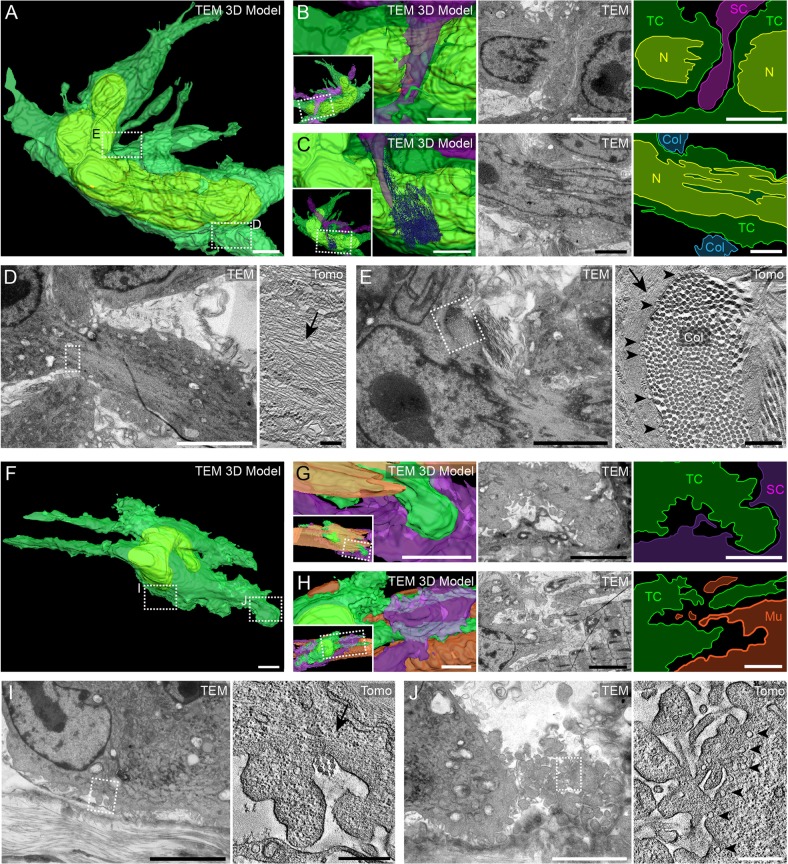
**Serial TEM and electron tomography analysis of tumor cell protrusions from subcutaneous xenografts.** (A) 3D model obtained from serial TEM imaging of the cell body of the tumor cell 1 highlighted in Fig. 7. The nucleus (yellow) and plasma membrane (transparent green) of the tumor cell are segmented. Dashed boxes indicate where the higher magnification images shown in D,E were obtained. Scale bar: 5 µm. (B,C) The tumor cell (TC) is flanked by one stromal cell (B, ‘SC’, purple) and a bundle of collagen fibers (C, Col, blue) at a position where the ultrastructure indicates that the nucleus (‘N’) is slightly confined. The left panels show zoomed views of the 3D model, its orientation is shown in the insets. The middle panels show TEM images, and the right panels show color-coded maps of the middle panels. Scale bars: 5 µm. (D,E) Electron tomography analysis of the boxed areas in A. Scale bars: 5 µm (left panels); 200 nm (right panels). Electron tomography imaging revealed the presence of multiple cytoskeletal filaments (arrows) in a protrusion (D) and underneath the plasma membrane at the cell body (E). Moreover, groups of vesicles associated with the plasma membrane (arrowheads) can be recognized in the region where the tumor cells are in close proximity with collagen fibers (Col), a feature that can be observed in multiple regions. (F) 3D model from serial TEM imaging of tumor cell 3 shown in Fig. 7. Dashed boxes indicate where the higher-magnification images shown in I,J were obtained. Scale bar: 10 µm. (G,H) The tumor cell and its cellular and acellular microenvironment. The left panels show zoomed views of the 3D model, the orientations are shown in the insets. The middle panels show TEM images, and the right panels show color-coded maps of the middle panels. Scale bars: 5 µm. (G) A rounded blebbing protrusion of the tumor cell (green) is in close proximity to a neighboring stromal cell (purple). Scale bars: 5 µm. (H) One side of the tumor cell (green) is in close apposition to the skeletal muscle cell (Mu, orange). Scale bar: 5 µm. (I,J) Electron tomography analysis of the boxed areas in F. Electron tomography images reveal the filament network (arrow) at the base of the bleb-like protrusion (I), collagen fibrils (Col, I) and small intracellular vesicles reminiscent of caveolae (arrowheads, J). Scale bars: 5 µm (left panels); 500 nm (right panels).

The resin-embedded sample was scanned by using microCT (Fig. 6D; Fig. S1). The microCT volume revealed several anatomical features that could also be seen using IVM, such as hair follicles, blood vessels and nerve fibers. The IVM and microCT volumes were segmented to create 3D models (Fig. 6E; Movie 8). The IVM model was docked into the microCT volume (Fig. 6F; Movie 8) revealing that the invasive protrusions were at a distance of 415 µm relative to the surface of the resin block (Fig. 6F, bottom panel). As described above (Fig. 2), knowing the position of the cells of interest within the resin block allowed rapid and accurate trimming towards the ROI. Then, a series of 330 sections (thicknesses of 130 nm) was collected, imaged at low-magnification with TEM and aligned (Fig. 7A; Movie 9). The resulting stack of TEM images was segmented to generate a 3D model of the invasive structures of the tumor cells and the associated vasculature (Fig. 7B). As predicted, the targeted invasive front of the tumor was located within the TEM *z*-stack, as shown by correlation between the electron microscopy and the IVM 3D models (Fig. 7B). Only a 4.2 µm difference was calculated between the actual and predicted position of the first targeted protrusion (Fig. 7, arrowhead 1). Based on the time-lapse IVM analysis of the dynamic invasive front, we identified and successfully retrieved two invasive cells with obvious morphological differences (Figs 6B and 7; Movie 7). The first cell displayed two very long filament-rich protrusions that extended deep into the tissue (Fig. 7A). The perinuclear region of the cell lay between collagen bundles as well as between neighboring stromal cells (Fig. 8A–E; Movie 9). The second invasive cell sat deeper in the tissue and lay on a skeletal muscle fiber (Fig. 7A). This cell showed obvious differences in terms of morphology of the protrusive structures (Figs 7A and 8F–J; Movie 9). Taken together, these data demonstrate that we can reliably and rapidly visualize dynamic events of tumor invasion and resolve them in TEM sections from the resin-embedded tissue.

### Zooming in on tumor cell protrusions and their micro-environment

Ultrastructural details of the two identified invasive cells (Fig. 7A, arrows 1 and 3) were imaged by using serial TEM and electron tomography (Fig. 8; Movies 9 and 10). Electron tomography analysis allows the visualization of fine ultrastructural features, such as cytoskeleton fibers, local extracellular matrix components and intracellular organelles (Fig. 8). Serial TEM and reconstruction of the first invasive cell showed the overall cellular and nuclear morphology (Fig. 8A–C). This invasive cell displayed a deformed nucleus that could result from a constriction between a neighboring stromal cell and a collagen bundle (Fig. 8A–C; Movie 10). With electron tomography, we then zoomed further into subcellular regions of this invasive cell and observed that protrusions were enriched with cytoskeletal filaments (Fig. 8D). Electron tomography analysis of a perinuclear region close to a collagen bundle revealed a similar abundance of cytoskeletal filaments as well as intracellular vesicles, suggesting this region of the cell might react to some physical constraint that is imposed by the collagen bundle (Fig. 8E). These observations are in good agreement with recent findings (Wolf et al., 2013), suggesting that deformability of the nucleus is a key determinant of tumor cell invasion. A second cell was found to be intercalated between a skeletal muscle fiber and stromal cells (Fig. 8F–H). Although emanating from the same invasive front, this cell differed from the previous as it displayed several bleb-like protrusions all over its surface. As previously described *in vitro* (Charras and Paluch, 2008), electron tomography analysis revealed that these structures (Fig. 8I) displayed filament-free regions that contained, in some cases, many intracellular vesicles reminiscent of caveolae (Fig. 8J). Similar to the cell shown in Fig. 8A–E, close contacts with collagen bundles were observed (Fig. 8I). These results demonstrate the capacity of multimodal correlative imaging to resolve subcellular features of the dynamic and plastic processes of tumor invasion in native tissues.

## DISCUSSION

Here, we report an efficient, rapid and reproducible approach for 3DEM imaging of tumor cells at crucial stages of the metastasis cascade, as determined by using IVM. Our multimodal correlative microscopy integrates microCT to accurately predict the position of *in vivo* imaged single cells inside large resin blocks. This enabled the routine retrieval of single tumor cells (Figs 3 and 7; Fig. S4) and their sub-cellular structures (Figs 4, 5 and 8) for 3DEM analysis in tissue samples. In this study, we show the results from two different regions in one mouse brain and from one region in a mouse ear skin sample.

Our approach combines well-established imaging modalities and sample preparation techniques. As a consequence, it suffers from the limitations brought by each one of them. Intravital imaging, for example, relies on the imaging of fluorescently labeled structures, and thus requires the introduction of dyes or genetically encoded fluorescent proteins. Xenografts of genetically engineered fluorescent cells can be extremely efficient tools to accurately mimic patho-physiological situations, but these models can be a source of artifacts. Furthermore, preparing the samples for electron-microscopy-mediated visualization requires dedicated protocols. High-pressure freezing is the method of choice to preserve the ultrastructure of the sample, but this is currently only applicable to small samples (thickness of 200 µm or less). Large samples, such as whole organisms, require chemical fixation, but it is known that such fixation introduces structural artifacts into the sample (Korogod et al., 2015).

### Intravital multimodal correlative microscopy provides unique insights into metastatic processes

Linking functional IVM analysis of tumor cells, and their microenvironment, to high-resolution imaging with electron microscopy will contribute to the understanding of the various cellular processes that lead to the spread of cancer. Using this workflow, we observed important sub-cellular features of arrested tumor cells. For example, in tumor cells that had been trapped in a blood vessel, we observed cellular extensions towards the vascular wall (Fig. 4). These observations suggest that tumor cells use active cellular protrusions to cross the physical barrier imposed by the endothelium and basal lamina. The protrusions could not be resolved through intravital imaging in the mouse brain (Movie 1) owing to the limited resolution of IVM. The formation of cellular extensions and the observed drastic remodeling of the endothelial layer close to the tumor cell (Fig. 5) are potentially the earliest steps in extravasation. Recently, invadopodia have been identified as key players in tumor cell extravasation in the chicken chorioallantoic membrane model (Leong et al., 2014).

Electron tomography analysis of invading tumor cells revealed protrusions that were massively populated with bundles of filaments, and we showed their tight interactions with the local extracellular matrix (Fig. 8). Furthermore, in the migrating tumor cells, we observed a clear deviation of the normal spindle-shaped morphology of the nuclei (Fig. 8A–C,D,F). Other studies (Wolf and Friedl, 2011) have elegantly shown that cell migration through interstitial tissues is mostly controlled by the architecture of the extracellular matrix, in particular the matrix pore size. The nuclear deformability of cells can compensate for the small pore size and thus regulate the invasion abilities of cells (Friedl et al., 2011).

These observations confirm that this workflow has great potential in unraveling metastatic events at the nanoscale.

### Practical considerations of intravital multimodal correlative microscopy

Although the presented workflow substantially speeds up the correlation between IVM and electron microscopy analysis, obtaining a significantly relevant number of observations still demands several months of work. To reveal the structural changes that a cell undergoes during extravasation, for example, might require 20 to 30 independent experiments. We estimate that such a study would take at least 7 months of full-time work for a single person, taking into account that some steps in the workflow can be performed in parallel. We believe, however, that for many *in vivo* studies, the unique gain in resolution will be worth the investment of time. Importantly, every single tumor cell (*n*=6, four are shown here) that has been targeted so far was successfully retrieved and imaged by using electron microscopy, illustrating the high success rate of the approach.

The multimodal correlative workflow requires access to a set of high-end equipment in order to perform the described analyses (intravital microscopy, microCT scanning, TEM tomography and/or FIB-SEM). After the intravital imaging, however, the sample is fixed and can be sent to collaborating laboratories where specific imaging could be performed. Therefore, if a laboratory does not possess the whole set of equipment, as in our case, this workflow is still achievable by establishing collaborations with other research groups or industry.

### Intravital multimodal correlative microscopy – other approaches and applications

Studying complex 3D samples with electron microscopy requires knowing the position of the ROI in the *x*, *y* and *z* planes. In order to identify the ROI within resin blocks, others have preserved the signal of fluorescent markers during electron microscopy processing (Lucas et al., 2012; Nixon et al., 2009). However, weak fluorescence signal is quenched when conventional electron microscopy processing methods are used. Moreover, the limited imaging depth and *z*-plane resolution of fluorescence microscopy hinders accurate visualization of the ROI inside the resin block. The approach presented here does not require a compromise in the electron microscopy processing protocols; by registering the IVM volume into the microCT scan of the processed sample, we could predict the position of our target with accuracy of <5 µm.

MicroCT imaging of resin-embedded samples has been used in previous studies to identify a ROI inside the resin block, in order to specifically target this area using electron microscopy (Burnett et al., 2014; Sengle et al., 2013). In these studies, however, the targeted ROIs were either clearly visible in the microCT scans or revealed by using electron-dense markers (Bushong et al., 2015). The former approach is therefore restricted to rather large and contrasted features, whereas the latter approach relies on photo-oxidation or an enzymatic reaction. This leads to dense precipitates (e.g. DAB) that mask the ultrastructure of the targeted cells. Our approach, however, predicts the position of the target without requiring its direct visualization by using microCT. Here, for the first time, microCT was employed to bridge IVM to electron microscopy, and 3D registration was performed to retrieve an *in vivo* observed event in a resin-embedded sample.

Other correlative methods have successfully targeted individual synapses within the mouse brain by introducing artificial landmarks with laser branding (Bishop et al., 2011; Maco et al., 2013). In this powerful approach, the retrieval of the ROI in the *x*, *y* and *z* planes solely relies on the positioning of the NIRB mark in the tissue – i.e. the branding was performed either right above or in the same plane as the ROI. NIRB efficiency, however, is highly sensitive to the scattering of light in deeper regions of the tissue. Therefore, NIRB can only be performed on 60- to 100-µm vibratome tissue sections that contain the ROI. Our method skips obtaining and screening such sections, and can address bulkier tissue pieces. We believe, therefore, that our approach can provide a complementary yet more versatile way to correlate intravital imaging with ultrastructure.

In conclusion, we have established a versatile and precise multimodal imaging approach that allows efficient correlation of *in vivo* imaging with volume electron microscopy. Applied here to cancer biology, this workflow has already provided informative and unexpected observations of the metastatic behaviors of single tumor cells within realistic pathological situations. We demonstrated our multimodal correlative imaging approach on mouse brain and skin tissue, but the workflow can be applied to many other cell biology events that can be imaged *in vivo*. The enhanced throughput of the method will allow routine use in translational research on animal models and holds great potential for understanding multiple biological processes at the ultrastructural level.

## MATERIALS AND METHODS

### Mouse handling

#### Mounting the cranial window, intracardiac injection of tumor cells and anesthesia

To perform intravital imaging of the cerebral cortex, a cranial window was grafted onto an 8–10-week-old immunodeficient female mouse (*Mus musculus*) (NU/NU nude mouse, Charles River Laboratories International, Sulzfeld, Germany), as previously described. After a 3-week healing period, the mouse received a 100 µl solution of cytoplasmic GFP-expressing JIMT1 cells (1×10^7^ cells/ml in PBS) through intracardiac injection through the left ventricle. Intravital imaging was performed immediately following injection, and on the second and third day post-injection. For imaging, the mouse was anesthetized by using isoflurane. To visualize the blood vessels, 100 µl of TRITC–dextran (500 kDa, Sigma-Aldrich) was administered to the mouse through injection into the tail vein. The mouse was placed on a custom-made holder (Deutsches Krebsforschungszentrum, Heidelberg, Germany) and remained anesthetized by using an isoflurane gas anesthesia system. All animal experiments were conducted in agreement with the regulations for animal experimentation dictated by the state Baden-Württemberg (Germany), and were approved by the state authorities.

#### Subcutaneous injection of tumor cells

An immunodeficient adult 8- to 10-week-old mouse (Rj:NMRI-Foxn1nu/Fox1nu, Janvier labs, Saint-Berthevin, France) was injected with the D2A1 Lifeact-YPet cell line, as previously described (Karreman et al., 2014).

### Two-photon excitation IVM and NIRB

#### IVM and NIRB through the cranial window

The mouse was mounted on an upright confocal microscope (Zeiss LSM 7, controlled by the software Zen 2012, Carl Zeiss Microscopy GmbH, Jena, Germany) and kept at a steady temperature of 32°C by means of a heating pad. The holder kept the mouse head immobile and allowed analysis of the same imaging area over days by keeping track of the *x*,*y* coordinates of the microscope stage. IVM was performed at an excitation wavelength of 850 nm (680–1080 nm Chameleon Ultra II laser, Coherent, Santa Clara, CA) using a ×20 Zeiss W Plan Apochromat N.A. 1 objective (Carl Zeiss Microscopy GmbH). The GFP and TRITC emission wavelengths (510 and 573 nm, respectively) were collected by two hybrid non-descanned detectors. As the perfusion fixation leads to a wash out of the TRITC–dextran, fluorescent lectin from *Lycopersicon esculentum* (Sigma-Aldrich Chemie GmbH, Munich, Germany) was injected into the mouse tail vein in order to record the 3D organization of the blood vessels after fixation. The mouse was perfusion-fixed through intracardiac injection with 2.5% glutaraldehyde (Electron Microscopy Sciences, Hatfield, PA) and 2% formaldehyde (Electron Microscopy Sciences) in 0.1 M PHEM buffer (comprising 60 mM PIPES, 25 mM HEPES, 10 mM EGTA and 2 mM MgCl, pH adjusted to 6.9). Following fixation, the ROI was imaged again based on the stored stage *x*,*y*-coordinates. NIRB was performed with the same laser that had been used for IVM, tuned to a 750 nm wavelength. Above the ROIs, at the level of the brain surface, a 150×150 µm^2^ area was scanned in a single focal plane until the NIRB square became clearly visible through emission of autofluorescence in the green channel. Around the ROI, three bigger 300×300 µm^2^ NIRB squares were drawn in non-symmetric positions to facilitate orientation and retrieval of the ROI upon dissection (Fig. 2). The brain was removed from the skull and post-fixed by immersion in the same fixative at 4°C overnight. The following day, the fixative was replaced with 0.1 M PHEM buffer, and the brain was stored at 4°C until further processing.

#### IVM and NIRB analysis of subcutaneous tumors in the mouse ear

IVM and NIRB were performed as previously described (Karreman et al., 2014) on the anesthetized mouse. After the IVM data acquisition, the mouse was fixed through intracardiac perfusion of 2.5% glutaraldehyde and 2% formaldehyde in 0.1 M PHEM buffer. The ear was dissected and immersed in the same fixative at 4°C overnight. The next day, the ear biopsy was transferred to 0.1 M PHEM buffer and stored at 4°C until further processing.

### Processing for electron microscopy

To improve the penetration of the chemicals during processing, small samples (<1 mm^3^) needed to be selected from the large brain and ear biopsies. Fixation and storage had not influenced the visibility of the NIRB markings on the surface, and small samples could be dissected around these square shapes. The samples were processed in a PELCO Biowave Pro microwave (Ted Pella, Redding, CA), using a protocol based on previous work (Cantoni et al., 2010). The samples were washed four times, 5 min each in cacodylate buffer (pH 7.4), in a laminar flow hood. Primary post-fixation with 1% OsO_4_ (Electron Microscopy Sciences, Hatfield, PA) and 1.5% K_4_Fe(CN)_6_ (Merck) in 0.1 M cacodylate buffer was performed in the microwave under a vacuum in seven consecutive 2-min steps, cycling between 100-W power on–off stages. The samples were then rinsed thoroughly in the laminar flow hood and twice for 40 s at 250 W in the microwave. Secondary post-fixation with 1% OsO_4_ in 0.1 M cacodylate buffer was then performed under the same conditions as described for the primary post-fixation. Staining with 1% uranyl acetate (Serva Electrophoresis GmbH, Heidelberg, Germany), dehydration in ethanol and resin embedding were performed in the microwave, as described previously (Karreman et al., 2014). The mouse brain samples were mounted in commonly used resin molds and left to polymerize for 3 to 4 days at 60°C. The ear skin tissue was flat embedded between two sheets of aclar and mounted on an empty resin block the following day, as described before previously (Karreman et al., 2014). Following polymerization, the front face and the sides of the resin blocks were trimmed using a trimming diamond knife (20°, Diatome AG, Biel, Switzerland) to create a reference surface for future measurements (see text and below).

### MicroCT imaging and reconstruction

MicroCT scanning was performed with a Phoenix Nanotom m (GE Sensing & Inspection Technologies, Fairfield, CT) operating under xs control and Phoenix datos|x acquisition software (both GE Sensing & Inspection Technologies). The resin-embedded biopsies were cut from the large resin block to achieve a small (<3 mm^3^) sample. This allowed mounting of the sample as close as possible to the X-ray source, which resulted in a higher magnification and resolution upon imaging. Scanning of the brain samples was performed at 50 kV and 500 µA for 1 h (1440 frames, average of 2, exposure time of 1 s). The skin sample was scanned in similar conditions, but with higher image averaging, which resulted in noise reduction and extended the scan time to 2 h. The voxel size of the resulting datasets was 0.75 µm in the *x*, *y* and *z* planes for the brain samples and 0.77 µm in the *x*, *y* and *z* planes for the ear skin sample. Reconstruction of the microCT volume was performed using Phoenix datos|x reconstruction software (GE Sensing & Inspection Technologies), and the volume was then further processed with VGStudio MAX software (Volume Graphics, Heidelberg, Germany).

### 3D registration of the IVM and microCT volumes in Amira software

The *z*-stacks (Fig. S1) were loaded into the digital space of Amira (FEI Company, Hillsboro, OR) and semi-automatically segmented using ‘segmentation editor’. Hereto, pixels representing the features of interest were selected by global or local (using the ‘magic wand’) thresholding of gray values. Gaussian filtering and masking facilitated the automatic segmentation. The Amira feature ‘normalize image’ was used to minimize the effects of the intensity gradient in the dataset that had been caused by incomplete penetration of the heavy metals in deeper parts of the tissue. Segmentation artifacts were corrected by using manual segmentation.

3D surface models were generated from the label files and were simultaneously visualized in the Amira software. First, the IVM model was roughly fitted into the microCT model by manual displacement in 3D (rotation and translation). This first fitting helped to identify common features in both datasets. Second, using the ‘landmark (2 sets)’ module, the corresponding points in both datasets were selected (10–20 points). Using the ‘Landmarksurfacewarp’ module, the surface model of the IVM dataset was then warped into the microCT volume using rigid (affine) transformations. Generally, this first registration revealed more common points between both datasets, which could then be added to the ‘landmark set’, improving the final 3D registration.

Where the IVM dataset provided the position of the ROI with respect to the structural features within the biopsy, the microCT volume revealed the orientation of the biopsy inside the resin block (see text). By combining both datasets, the location of the ROI inside the resin block could thus be predicted. Using Amira, the distance between the ROI and the surface and sides of the resin block was measured.

### Trimming and approach at the ultramicrotome

The sample was mounted onto a Leica Ultracut S ultramicrotome (Leica Microsystems, Wetzlar, Germany) and trimmed using a 20° trimming diamond knife (Diatome AG, Biel, Switzerland). Before microCT scanning, a flat surface block face with parallel straight sides was trimmed. Following microCT and 3D registration of both datasets, the front face was trimmed to the calculated depth of the ROI. While trimming, the approach to the ROI could be monitored by obtaining a few thick sections (500 nm) from the sample. The sections were mounted onto a Superfrost ++ glass slide, stained with Toluidine Blue and imaged with light microscopy. The light-microscopy sections were correlated to the microCT virtual sections. To achieve this, an ‘obliqueslice’ was created in Amira software and aligned to be perfectly parallel with the trimmed block face in the microCT dataset, and thus also parallel to the trimming angle. The orientation of the ‘obliqueslice’ virtual sections then corresponded to the physical thick sections (500 nm) produced with the ultramicrotome.

It was determined, by visual inspection, which virtual section matched the best to the light-microscopy section. The position of this selected virtual section within the microCT dataset then enabled us to measure the distance between the newly trimmed surface and the ROI (Fig. S3). In practice, a small number of checkpoints are sufficient to obtain an accuracy of about 5 µm for the targeting. We have used from 2 to 5, evenly spread throughout the trimmed volume. Collecting more checkpoints might lead to a higher degree of accuracy but will also slow down the whole procedure.

For FIB-SEM imaging, both the front face and one flank of the block needed to be trimmed very close to the region of interest (see Results; Fig. S3). To achieve this, the front was trimmed to several micrometers (20–30 µm) just before reaching the tumor cell (as estimated from the microCT 3D map). Next, one flank was trimmed as close as possible to the tumor cell (maximum 20–30 µm), a new thick section was produced and compared to the microCT tomogram, to check the progression towards the ROI (Fig. S3). The front face, which would be the imaged surface in the FIB-SEM analysis, was then trimmed further to approach the cell as close as possible (3–5 µm). To enable mounting on the SEM stub, the trimmed tip of the resin block was cut away with a double-edged razor blade. The sample was mounted with an adhesive conductive carbon tab (PELCO Tabs, Ted Pella) onto a 0.5 ‘SEM pin stub’ (Agar Scientific, Essex, United Kingdom) and stabilized with colloidal silver (liquid, Ted Pella). The sample on the stub was then sputter-coated with gold (60 s at 30 mA).

For serial sectioning of the resin-embedded subcutaneous tumor sample, the front face of the resin block was trimmed to the calculated depth of the region of interest. The flanks of the samples were trimmed to create a block face of approximately 300×600 µm^2^. Approximately 330 thick serial sections (130 nm) were produced with a 45° histo diamond knife (Diatome AG, Biel, Switzerland) and mounted onto formvar-coated slot grids.

### Electron microscopy and image analysis

The mouse brain samples were imaged in an Auriga 60 FIB-SEM instrument (Carl Zeiss Microscopy GmbH) operating under SmartSEM (Carl Zeiss Microscopy GmbH) and Atlas3D software (Fibics Incorporated, Ottowa, Ontario, Canada). The resulting datasets were aligned using TrakEM (Cardona et al., 2012) [ImageJ (Schindelin et al., 2012); http://imagej.nih.gov/ij/], and the image stack was segmented in 3dmod, part of the IMOD software package (Mastronarde, 2005) (Boulder Laboratory, University of Colorado, Denver, CO). The serial sections of the mouse ear skin tissue were screened and imaged with a Biotwin CM120 instrument (120 kV, FEI Company, Hillsboro, OR) equipped with a bottom-mounted 1 K Keen View CCD camera (Olympus Soft Imaging Solutions, Münster, Germany). Montaged images and tomograms were acquired with a Tecnai F30 Field Emission Gun TEM (300 kV, FEI Company) equipped with an Eagle 4 K camera (FEI Company). The F30 was controlled using Tecnai User Interface (PEOUI) and Tecnai Acquisition (TIA) software. Tomograms and image montages were obtained using SerialEM software (Mastronarde, 2005), and the tomograms were reconstructed in eTomo, part of the IMOD software package (Kremer et al., 1996) (Boulder Laboratory). The electron microscopy images were processed using ImageJ (Schindelin et al., 2012) and Photoshop CS6 (Adobe). The figures were created in Illustrator CS6 (Adobe), and the supplementary material movies were made with Fiji (ImageJ), IMOD (Boulder Laboratory) and Amira (FEI Company).
